# Supplementary material for: Time-resolved dual transcriptomics reveal early induced Nicotiana benthamiana root genes and conserved infection-promoting Phytophthora palmivora effectors
Source: BMC Biol. 2017 May 11;15:39. doi: 10.1186/s12915-017-0379-1 (PMC5427549; doi:10.1186/s12915-017-0379-1)
Supplement: Supplementary file 3 — Benchmarking universal single-copy orthologues (BUSCO) genes missing from available Phytophthora genomes and transcriptomes. Figure S2. Amino acid sequence alignment of PLTG_13552 and P. infestans AVR3aEM. Figure S3. HxGPCExxxDD-containing P. palmivora secreted proteins. Figure S4. Number of differentially expressed genes (DEGs) between infection time points. Figure S5. Validation of dynamic behaviour of P. palmivora DEGs by qRT-PCR. Figure S6. PCR detection of REX1–4 effectors in P. palmivora isolates. Figure S7. Amino acid sequence logos for REX1–4 effectors. Figure S8. Habitus of N. benthamiana transgenics used in this study. Figure S9. Subcellular localisation of GFP:REX1–4 proteins in N. benthamiana leaves. Figure S10. Structure of pTrafficLights construct and secretion inhibition assays. Figure S11. Validation of N. benthamiana DEGs by qRT-PCR. Figure S12. Amino acid sequence alignment of TIPTOP and similar N. benthamiana sequences with A. thaliana prePIPL1, prePIP1 and prePIP2. Figure S13. Induction of TIPTOP promoter in response to biotic and abiotic stresses. (PDF 17445 kb) [file 12915_2017_379_MOESM3_ESM.pdf]

Time-resolved dual transcriptomics reveals early induced *Nicotiana benthamiana* root genes and conserved infection-promoting *Phytophthora palmivora* effectors

Evangelisti E, Gogleva A, Hainaux T, Doumane M, Tulin F, Quan C, Yunusov T, Floch K, Schornack S

Supporting Figures

Figure S1 – BUSCO genes missing from available *Phytophthora* genomes and transcriptomes

Figure S2 – Amino acid sequence alignment of PLTG_13552 and *P. infestans* AVR3a^{EM}

Figure S3 – HxGPCExxxDD-containing *P. palmivora* secreted proteins.

Figure S4 – Number of DEGs between infection time points.

Figure S5 – Validation of dynamic behavior of *P. palmivora* DEGs by qRT-PCR.

Figure S6 – PCR detection of REX1-4 effectors in *P. palmivora* isolates.

Figure S7 – Amino acid sequence logos for REX1-4 effectors.

Figure S8 – Habitus of *N. benthamiana* transgenics used in this study.

Figure S9 – Subcellular localisation of GFP:REX1-4 proteins in *N. benthamiana* leaves.

Figure S10 – Structure of pTrafficLights construct and secretion inhibition assays.

Figure S11 – Validation of *N. benthamiana* DEGs by qRT-PCR.

Figure S12 – Amino acid sequence alignment of TIPTOP and similar *N. benthamiana* sequences with *A. thaliana* prePIPL1, prePIP1 and prePIP2.

Figure S13 – Induction of TIPTOP promoter in response to biotic and abiotic stresses.

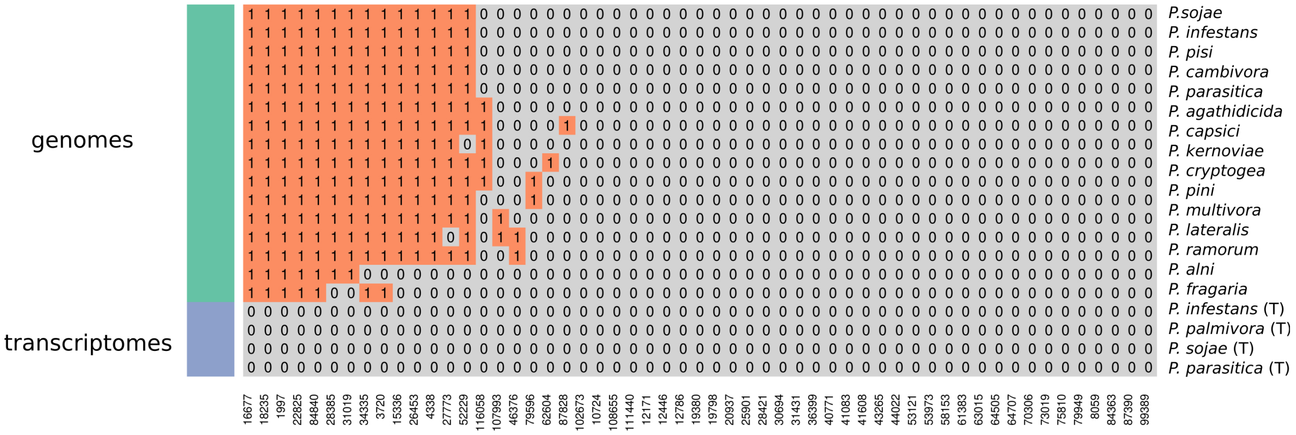

Figure S1 – BUSCO genes missing from available *Phytophthora* genomes and transcriptomes. ‘0’ represents missing BUSCO gene, ‘1’ represents a BUSCO gene present in the respective genome or transcriptome. Numbers on the x-axis correspond to actual identifiers of eukaryotic BUSCO genes used (BUSCO version 1).

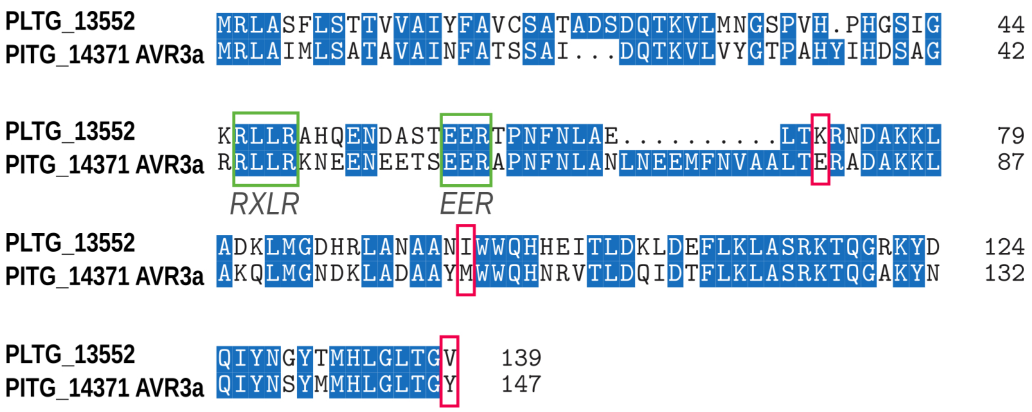

Figure S2 – Amino acid sequence alignment of PLTG_13552 and *P. infestans* AVR3a^{EM} (PITG_14371). Functionally assigned key residues in *P. infestans* AVR3a are framed in red: K/I80, I/M103 and terminal Y. Conserved RXLR and EER motifs are framed in green.

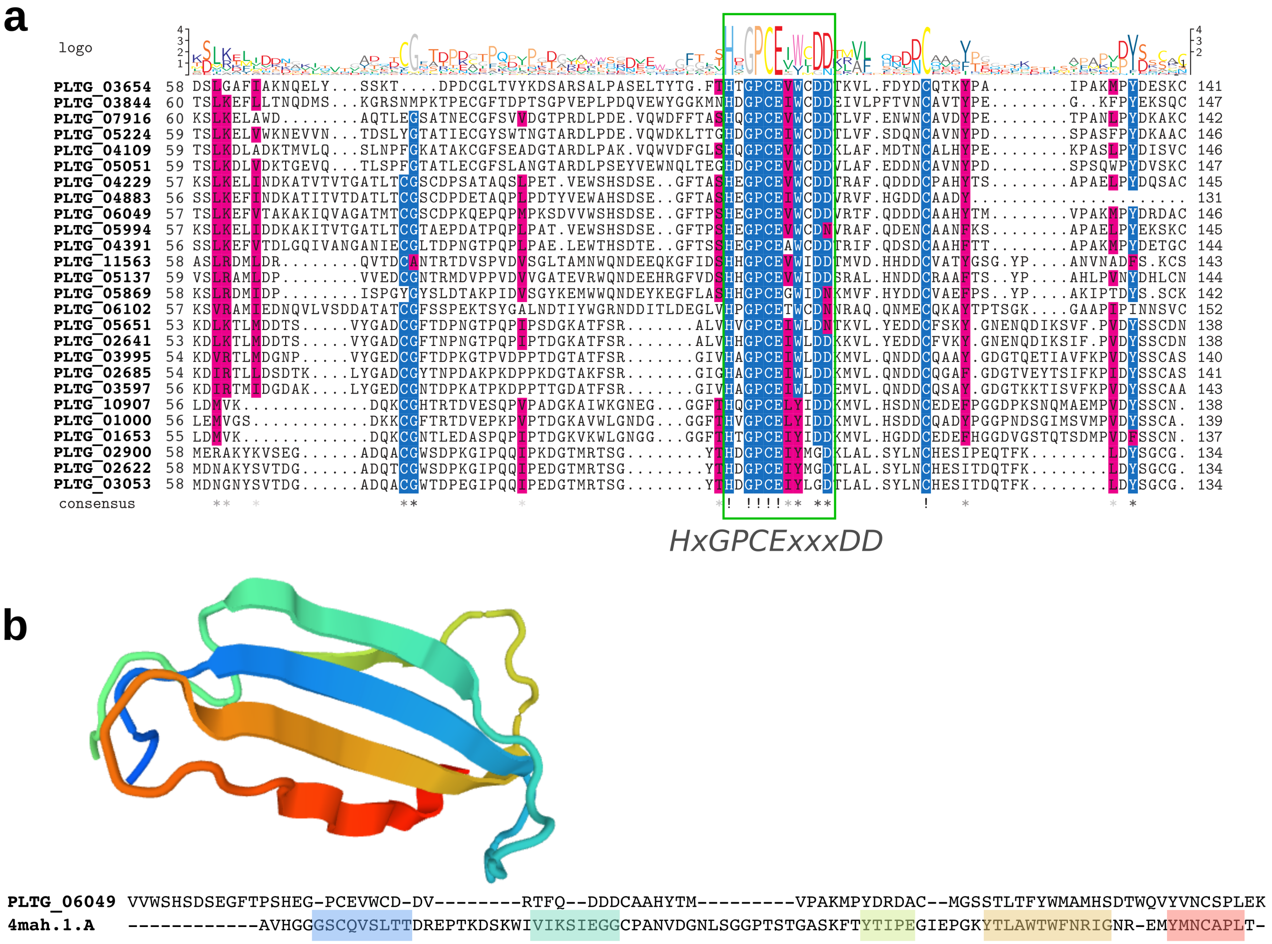

Figure S3 – HxGPCExxxDD-containing *P. palmivora* secreted proteins. (a) Amino acid sequence alignment of HxGPCE proteins. Alignment was performed on the full-length proteins and shows 60-160 positions of the N-termial domain alignment after signal peptide truncation. Conservative positions are shown in blue, similar positions are shown in magenta. **(b)** Homology modelling (SWISS MODEL) for PLTG_06049 protein showing partial structural similarity to AA11 lytic polysaccharide monooxygenase (4mah.1.A). Amino acids corresponding to the predicted β-sheets are shown in coloured blocks. GMQE (Global Model Quality Estimation) score = 0.5.

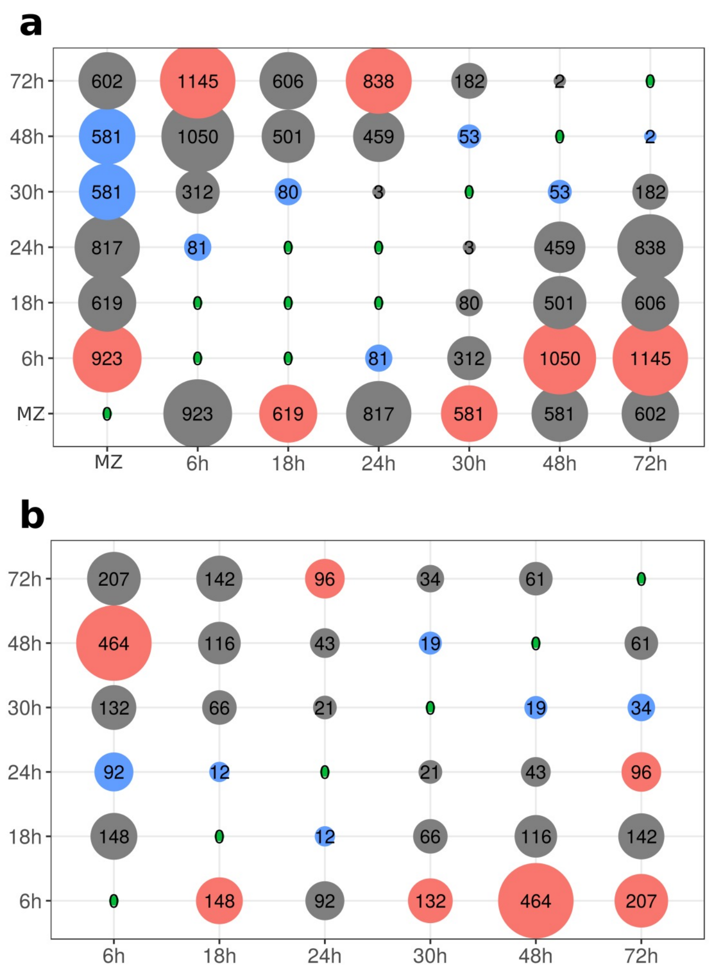

Figure S4 – Number of DEGs between infection time points. Numbers of differentially expressed genes between time points in pathogen **(a)** and plant **(b)** transcriptomes. The most different time point across all comparisons (*i.e.* within a column) is shown in red. The most similar time point across all comparisons (for positive numbers of DEGs) is shown in blue

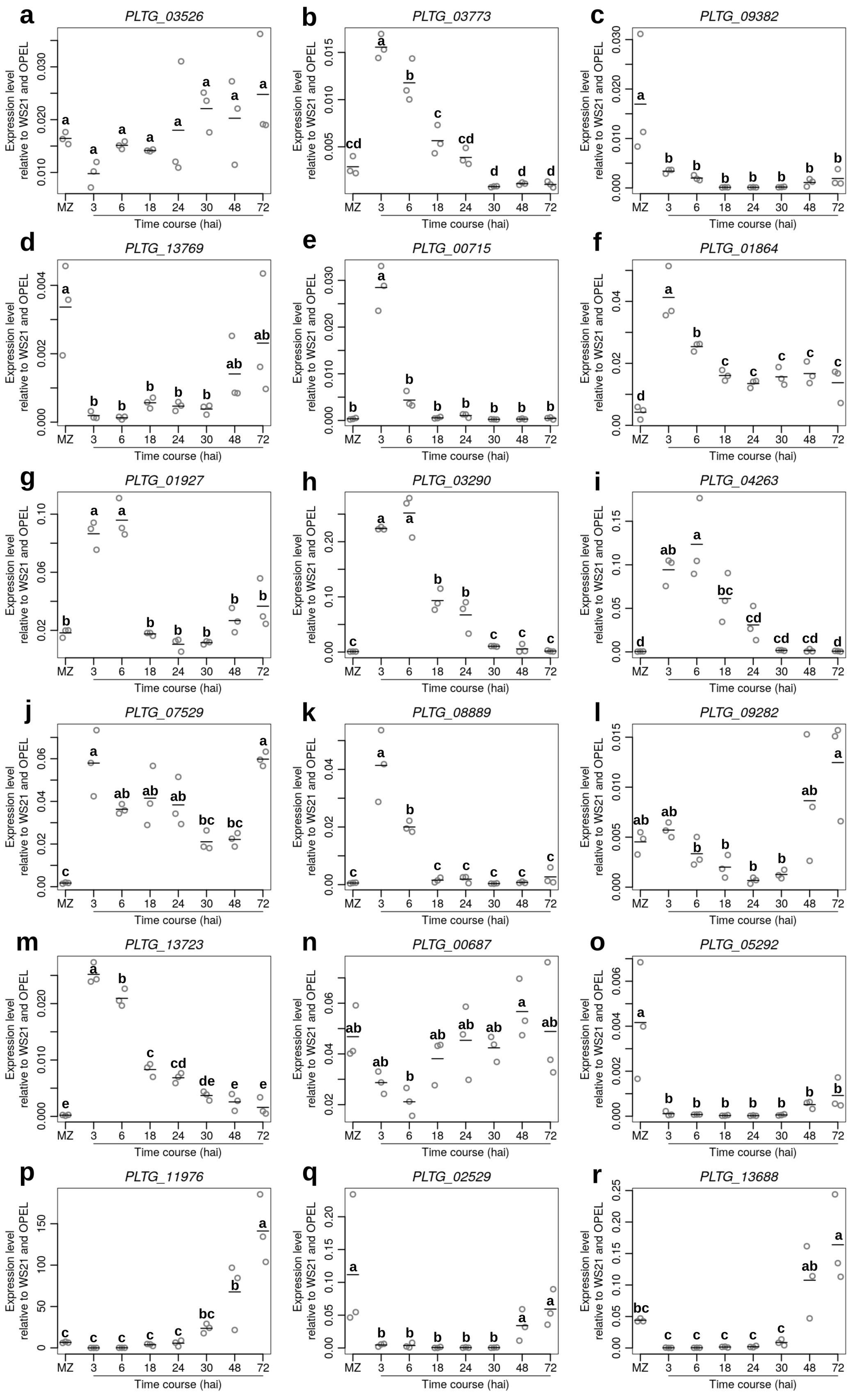

Figure S5 – Validation of dynamic behavior of *P. palmivora* DEGs by qRT-PCR. Accumulation of transcripts from eighteen *P. palmivora* genes from groups A **(a-d)**, B **(e-m)**, C **(n-p)** and D **(q, r)**. Expression profiles were determined *via* independent *N. benthamiana-P. palmivora* time course experiments. Values are given relative to *WS21* ad *OPEL* reference genes. Statistical significance was assessed using one-way ANOVA and Tukey's HSD test ($P < 0.05$). MZ : axenically grown mycelium with sporangia.

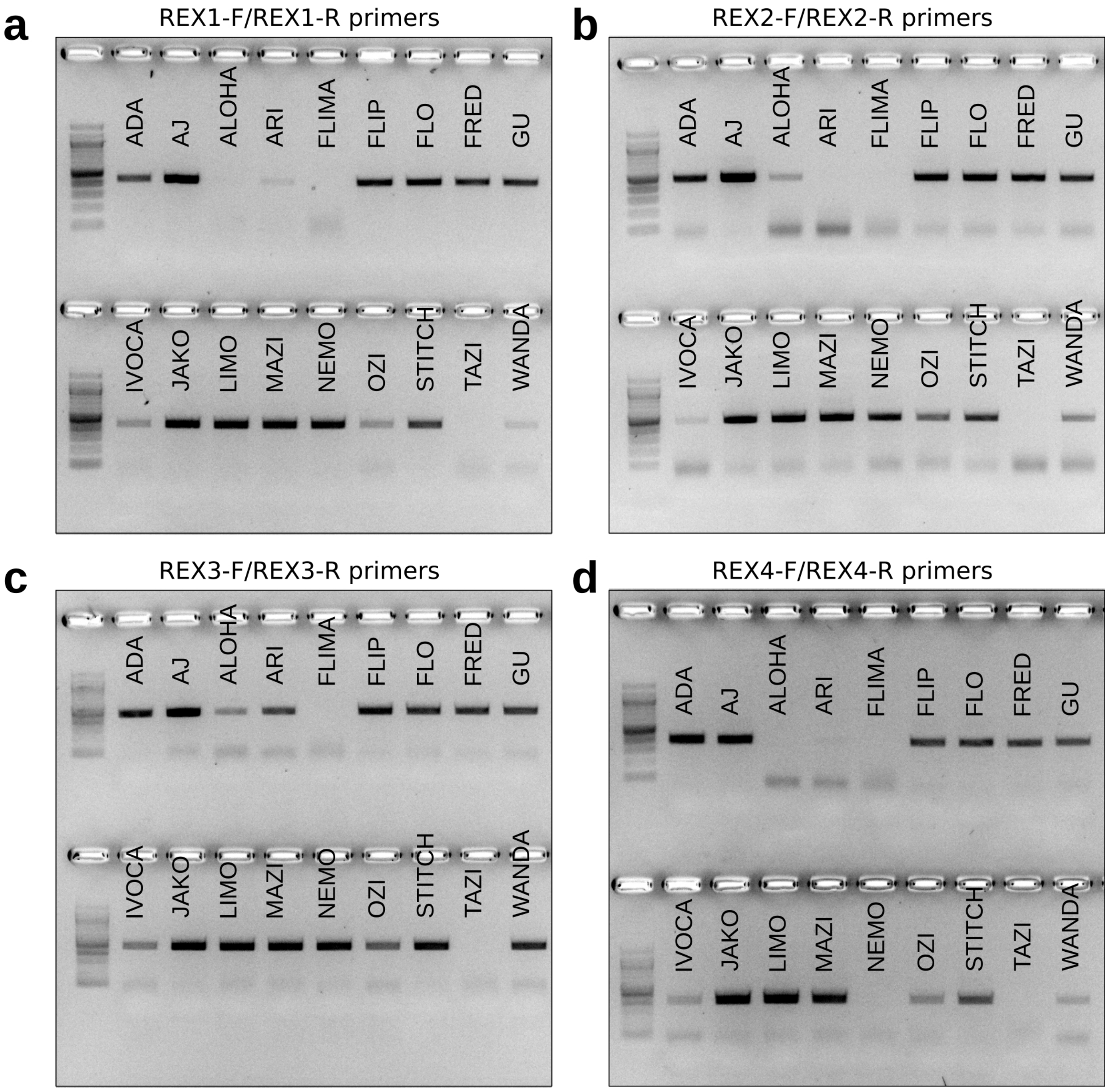

Figure S6 – PCR detection of REX1-4 effectors in *P. palmivora* isolates. REX1-4 coding sequences were amplified from genomic DNA extracted from axenically grown mycelium. Nucleotide sequences of the amplicons were confirmed by Sanger sequencing.

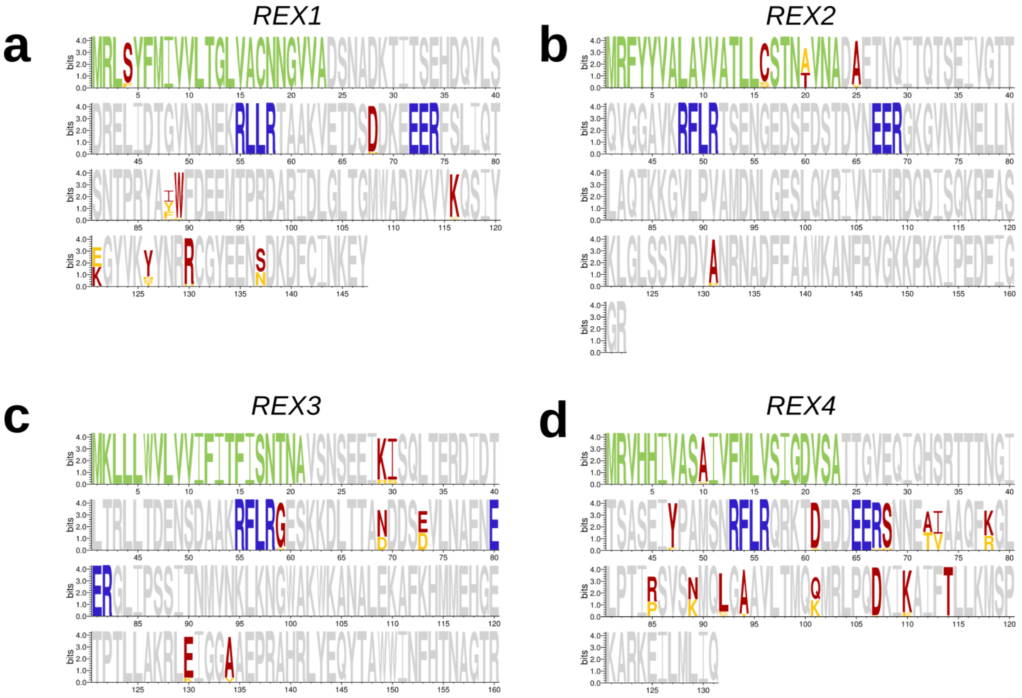

Figure S7 – Amino acid sequence logos for REX1-4 effectors. Amino acid variability of REX genes was assessed on 18 *P. palmivora* isolates. Conserved residues are shown in grey. Polymorphism is shown in yellow. Amino acids corresponding to the reference sequence used in this study (*P. palmivora* LILI strain) are shown in red. Signal peptide region is highlighted in green. Conserved RxLR and dEER motifs are shown in blue.

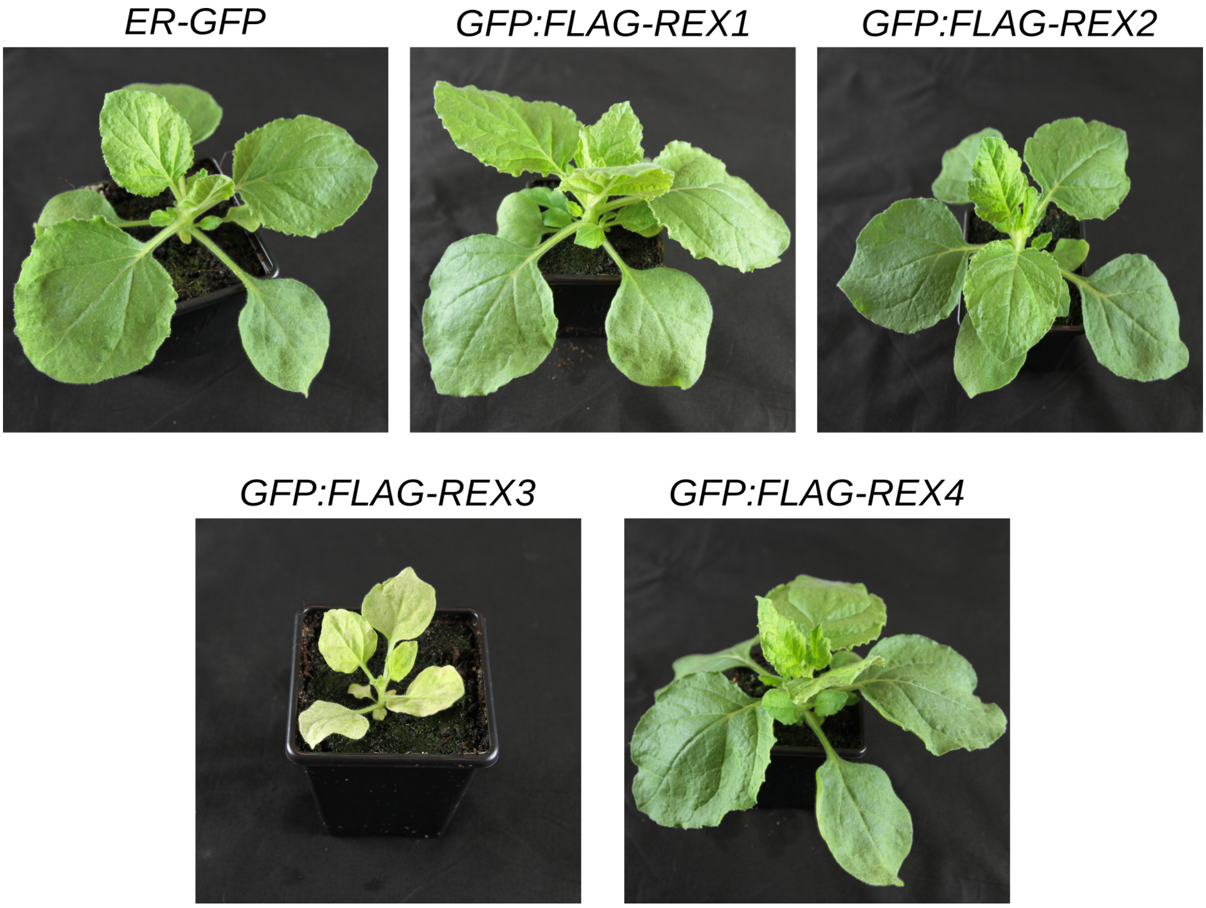

Figure S8 – Habitus of *N. benthamiana* transgenics used in this study. Representative pictures of transgenic *N. benthamiana* expressing ER-targeted GFP (GFP16c) used as control, GFP:FLAG-REX1, GFP-FLAG-REX2, GFP:FLAG-REX3 and GFP:FLAG-REX4.

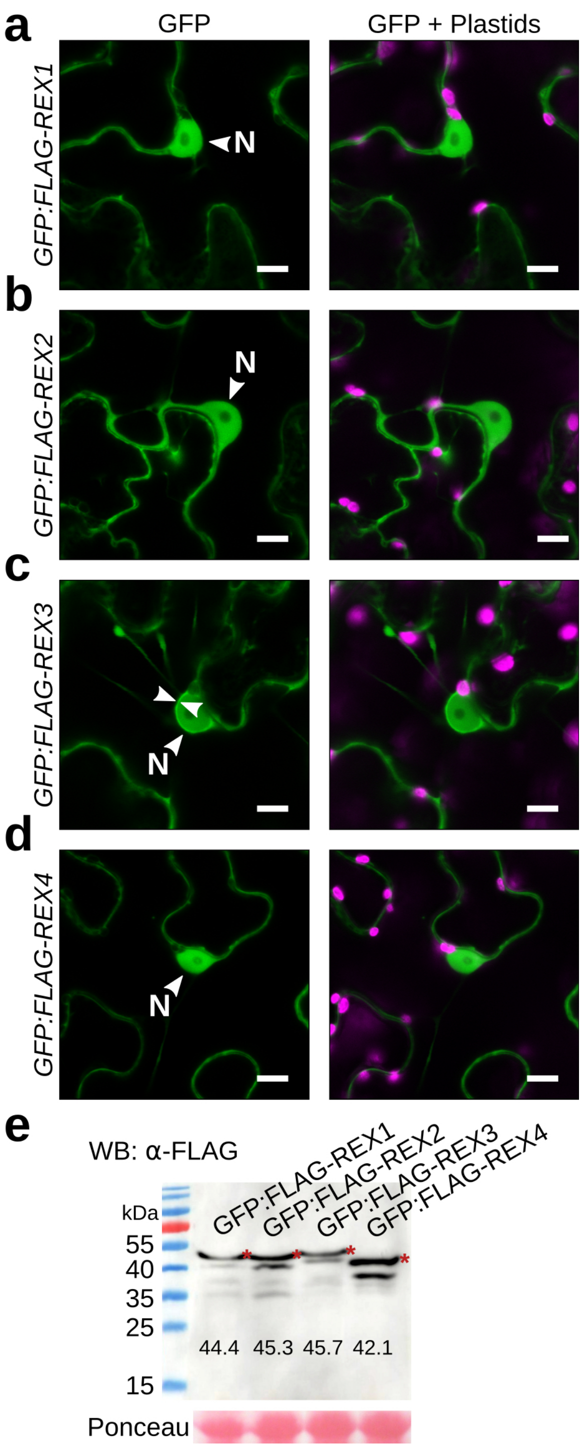

Figure S9 – Subcellular localisation of GFP:REX1-4 proteins in *N. benthamiana* leaves. (a-d) *Agrobacterium tumefaciens* cells carrying GFP:REX1-4 fusions were infiltrated in *N. benthamiana* leaves. GFP fluorescence was monitored 24 hours after agroinfiltration. GFP:FLAG-REX1 **(a)**, GFP:FLAG-REX2 **(b)** and GFP:FLAG-REX4 **(d)** accumulated in the cytoplasm and the nucleus, while GFP:FLAG-REX3 **(c)** accumulated preferentially in the cytoplasm and around the nucleus (N) (arrowheads). Scale bars are 10 µm. **(e)** Western Blot analysis of GFP:FLAG-REX protein degradation from *N. benthamiana* total leaf protein samples. Protein detection was achieved using anti-FLAG antibodies. Ponceau red staining is given as a control for protein loading. ProtParam predicted protein weight (kDa) is shown below the bands.

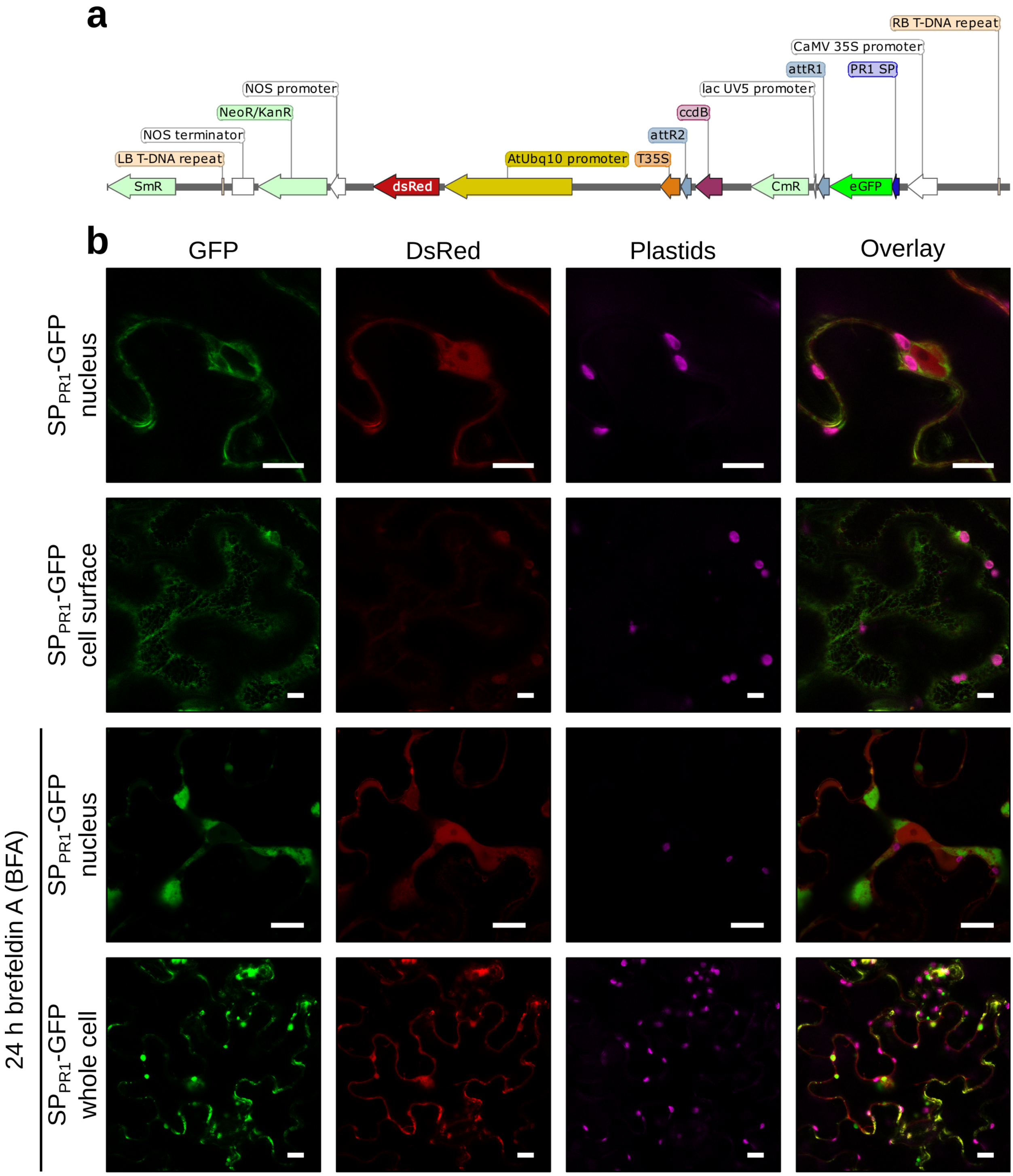

Figure S10 – Structure of pTrafficLights construct and secretion inhibition assays. (a) Secretion inhibition assays were performed using a pTrafficLights vector derived from the Gateway vector pK7WGF2. The signal peptide (SP) of PATHOGENESIS-RELATED 1 (PR1) was cloned in frame with the N-terminal GFP cassette. In addition, a AtUBQ10*pro*::DsRed cassette for constitutive DsRed expression (Limpens *et al*, 2004) was inserted within the T-DNA. **(b)** *Agrobacterium*-mediated transient expression of pTrafficLights in *N. benthamiana* leaves results in a weak labelling of the nucleus periphery and the endoplasmic reticulum. Secretion inhibition was confirmed by assessing GFP accumulation in aggregates after 24h treatment with brefeldin A (BFA). Scale bars represent 10 µm.

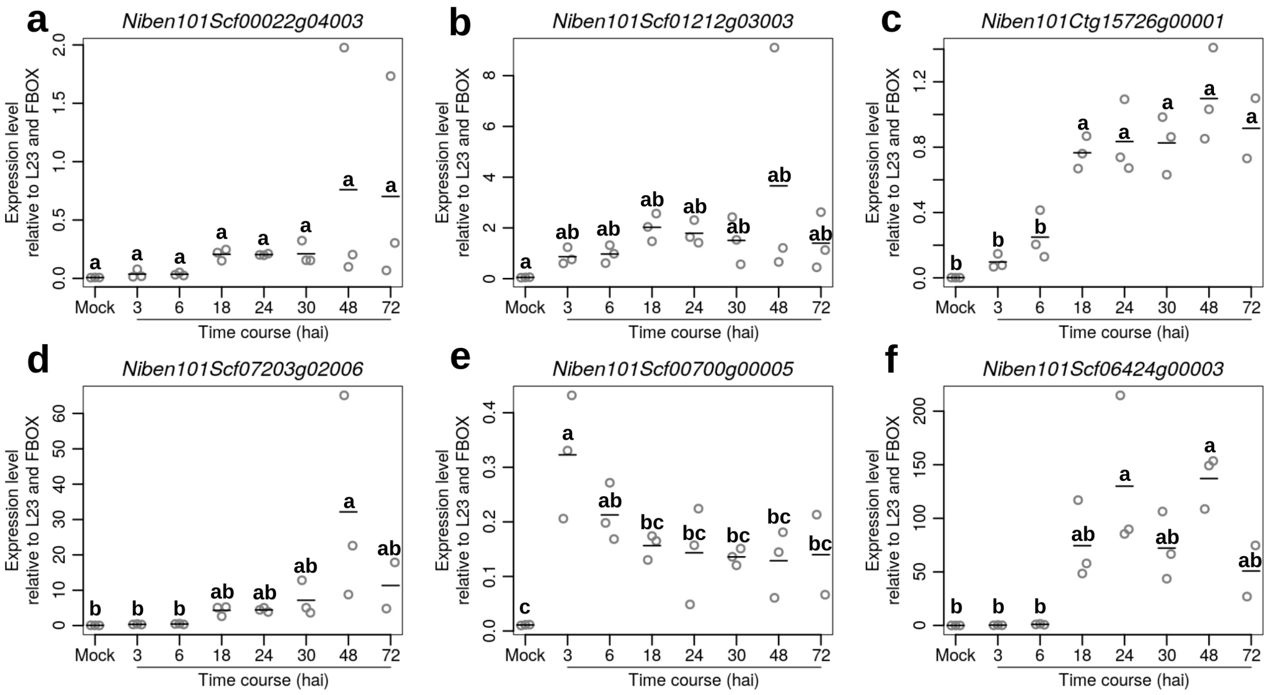

Figure S11 – Validation of *N. benthamiana* DEGs by qRT-PCR. Accumulation of transcripts from six genes upregulated upon *P. palmivora* infection was carried out by qRT-PCR. Expression profiles were determined through independent *N. benthamiana*-*P. palmivora* time course experiments. Values are given relative to *L23* ad *FBOX* reference genes. Statistical significance was assessed using one-way ANOVA and Tukey's HSD test ($P < 0.05$).

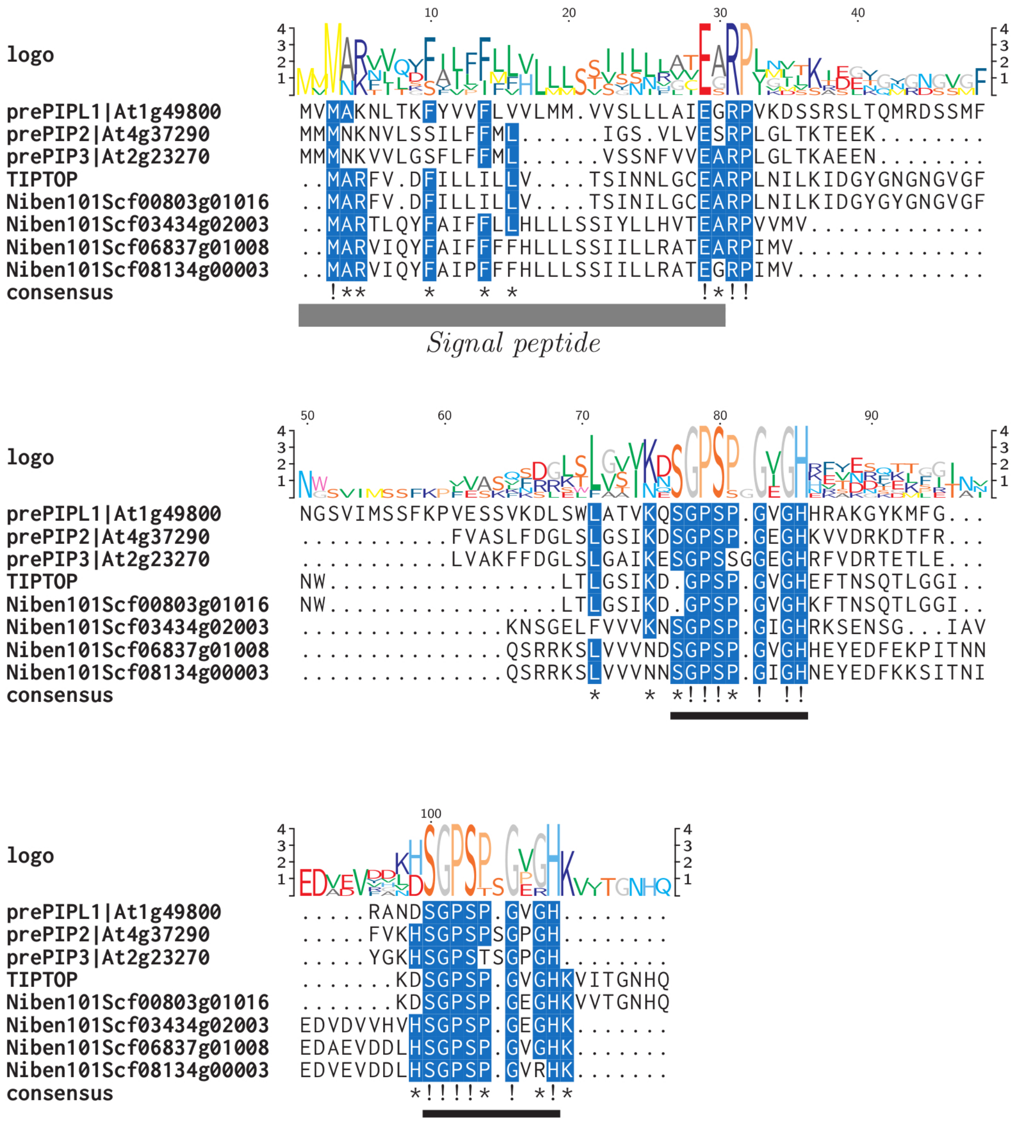

Figure S12 – Amino acid sequence alignment of TIPTOP and similar *N. benthamiana* sequences with *A. thaliana* prePIPL1, prePIP1 and prePIP2. Alignment was performed using TIPTOP (Niben101Scf03747g00005), four closely related peptide sequences (Niben101Scf00803g01016, Niben101Scf03434g02003, Niben101Scf06837g01008 and Niben101Scf08134g00003) and *A. thaliana* peptides prePIPL1 (At1g49800), prePIP2 (At4g37290) and prePIP3 (At2g23270) identified by Hou *et al* (2014). The eight sequences share a conserved arginine motif immediately following the signal peptide and two tandem repeats of a SGPS and GxGH motif in their C-terminal region.

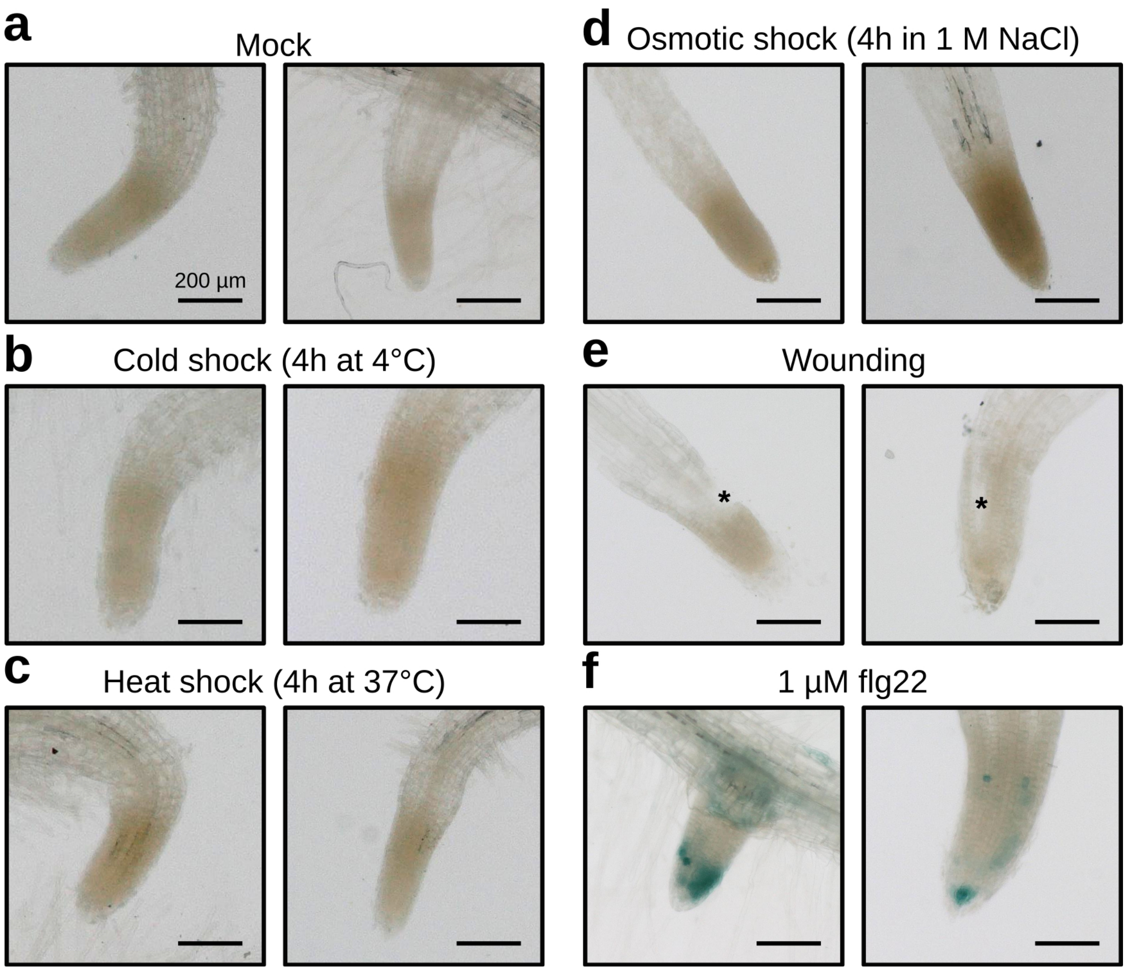

Figure S13 – Induction of TIPTOP promoter in response to biotic and abiotic stresses. Transgenic *N. benthamiana* roots carrying a TIPTOPpro::GFP:GUS reporter were incubated for 4 hours at 24°C (mock, **a**), 4°C (cold shock, **b**), at 37°C (heat shock, **c**), at 25°C in presence of 1 M sodium chloride (osmotic shock, **d**), wounded with a needle (**e**; *: wounded sites) or incubated with 1 µM bacterial flagellin for 4 h (flg22; **f)** prior to GUS staining. Scale bar represent 200 µm.

Supporting Methods

PCR amplification of effectors from *P. palmivora* isolates

Presence of the four selected *P. palmivora* effectors was assessed by PCR amplification of genomic DNA from isolates harvested in Australia (OZI P6269), Indonesia (JAKO P3738, AJ P6390), Malaysia (MAZI P6375, WANDA P6972), Philippines (FLO P7536, FLIMA P7545, FRED P7547, FLIP P7548, ADA P7551), Thailand (TAZI P6802), India (NEMO P0633), Sri Lanka (LIMO P6276), USA (ARI P3914) and USA Islands (Hawaii: ALOHA P6053, STITCH P0113; Guam: GU P11004). Genomic DNA was extracted as described by Möller *et al* (1992). An aliquot (20 ng) was used to perform 30-cycle amplification of effector coding sequence using Phusion DNA polymerase (Promega, USA) and primers shown in **Table S3**. Amplification was confirmed by electrophoresis on 2 % TAE-agarose gel and amplicons were subsequently sequenced (Source Biosciences, UK).

Western Blot analysis

Absence of degradation of the full length GFP:FLAG-REX1-4 fusion proteins was confirmed by Western Blot using agroinfiltrated leaf tissues. Leaf discs were flash-frozen in liquid nitrogen and ground with a TissueLyser (Qiagen, Hilden, Germany). Total proteins were extracted in GTEN buffer (10 % glycerol, 25 mM Tris-HCl, 1 mM EDTA, 150 mM NaCl) containing 0.1 % Tween-20. Samples were then submitted to SDS-PAGE and transferred onto nitrocellulose membranes. The quality of the transfer was assessed by Ponceau Red staining. Membranes were then blocked in TBS containing 2% skimmed milk. Membranes were incubated with monoclonal rabbit anti-FLAG antibodies (Sigma-Aldrich) at 1:1000 dilution. After washing, membranes were incubated with horseradish peroxidase (HRP)-coupled goat anti-Rabbit IgG antibodies (Sigma-Aldrich) at 1:10000 dilution. Antibody binding was revealed by enhanced chemiluminescence ECL (Pierce, Rockford, IL) using an Odyssey CLx Imaging System (LI-COR Biosciences).
